# A Network Extension of Species Occupancy Models in a Patchy Environment Applied to the Yosemite Toad (*Anaxyrus canorus*)

**DOI:** 10.1371/journal.pone.0072200

**Published:** 2013-08-12

**Authors:** Eric L. Berlow, Roland A. Knapp, Steven M. Ostoja, Richard J. Williams, Heather McKenny, John R. Matchett, Qinghua Guo, Gary M. Fellers, Patrick Kleeman, Matthew L. Brooks, Lucas Joppa

**Affiliations:** 1 United States Geological Survey, Western Ecological Research Center, Yosemite Field Station, Oakhurst, California, United States of America; 2 Vibrant Data Labs, Berkeley, California, United States of America; 3 Sierra Nevada Aquatic Research Laboratory, University of California, Mammoth Lakes, California, United States of America; 4 Microsoft Research, Cambridge, United Kingdom; 5 Quid Inc., San Francisco, California, United States of America; 6 National Park Service, Yosemite, California, United States of America; 7 University of California, Merced, California, United States of America; 8 United States Geological Survey, Western Ecological Research Center, Pt. Reyes Station, California, United States of America; University of Alberta, Canada

## Abstract

A central challenge of conservation biology is using limited data to predict rare species occurrence and identify conservation areas that play a disproportionate role in regional persistence. Where species occupy discrete patches in a landscape, such predictions require data about environmental quality of individual patches and the connectivity among high quality patches. We present a novel extension to species occupancy modeling that blends traditional predictions of individual patch environmental quality with network analysis to estimate connectivity characteristics using limited survey data. We demonstrate this approach using environmental and geospatial attributes to predict observed occupancy patterns of the Yosemite toad (*Anaxyrus* (= *Bufo*) *canorus*) across >2,500 meadows in Yosemite National Park (USA). *A* *. canorus* , a Federal Proposed Species, breeds in shallow water associated with meadows. Our generalized linear model (GLM) accurately predicted ~84% of true presence-absence data on a subset of data withheld for testing. The predicted environmental quality of each meadow was iteratively ‘boosted’ by the quality of neighbors within dispersal distance. We used this park-wide meadow connectivity network to estimate the relative influence of an individual Meadow’s ‘environmental quality’ versus its ‘network quality’ to predict: a) clusters of high quality breeding meadows potentially linked by dispersal, b) breeding meadows with high environmental quality that are isolated from other such meadows, c) breeding meadows with lower environmental quality where long-term persistence may critically depend on the network neighborhood, and d) breeding meadows with the biggest impact on park-wide breeding patterns. Combined with targeted data on dispersal, genetics, disease, and other potential stressors, these results can guide designation of core conservation areas for *A* *. canorus* in Yosemite National Park.

## Introduction

An important goal of conservation biology is to identify specific areas within a species’ range that play a disproportionately important role in the regional or global persistence of the species [1]. Important obstacles to identifying such “hotspots” include most generally that species often are patchily distributed, that these patches are dynamic in nature, and that we often have limited data on occurrence and spatio-temporal dynamics of the species.

There are many existing dynamic and static approaches to predict population hotspots for species with patchy distributions. Dynamic meta-population or patch occupancy models typically require detailed data on patch-specific colonization and extinction probabilities and dispersal functions (e.g., state transition models [2,3], and spatially realistic simulation models [4–6]), or complete occupancy data for many patches across large landscapes (e.g., incidence function models [7,8]). Unfortunately for most species, especially rare ones, these data are either sparse or unavailable. Alternative static statistical approaches model the probability of patch occupancy as a function of biotic and abiotic variables. These models can be parameterized with species presence/absence data collected over relatively short time periods [9], and they can incorporate uncertainly in occupancy due to imperfect detection [10,11]. The role of dispersal among patches can be incorporated by, for example, including the density of occupied patches within a defined radius or the distance to the nearest occupied patch [9]. This approach typically requires that all potential habitats in the study area be surveyed for the presence/absence of the target species and environmental characteristics (e.g., [9,12–14]). When developing occupancy models for large areas this requirement presents a formidable challenge. In sum, many approaches either require data with more resolution in time or space than is typically available for rare species over large areas, or they require enough data to accurately estimate patch-dynamic model parameters to avoid large error propagation [15].

Here we use a novel approach to static species occupancy modeling that allows us to include the potential dispersal network structure as a predictor despite having neither complete species presence/absence data for the entire study area, nor detailed time series data to estimate colonization/extinction rates. Our approach combines a static species distribution model with network analysis to infer patch connectivity and thereby estimates which patches are likely to have a disproportionate influence on the regional population. This network extension of a simple species occupancy model enables general inferences about the relative role of spatial ‘network quality’ (i.e., meadow neighborhood conditions conducive to colonization) versus intrinsic ‘environmental quality’ (i.e., coarse environmental attributes intrinsic to a meadow that are conducive to breeding) of habitat patches that are linked by dispersal.

We illustrate this approach with a case study of the Yosemite toad (*Anaxyrus* [*= Bufo*] *canorus*, hereafter *A* *. canorus* ). *A* *. canorus* is endemic to the Sierra Nevada mountains of California, and breeds in aquatic habitats associated with high elevation meadows. In our modeling, we used extensive (but not complete) toad presence/absence data collected across all of Yosemite National Park, USA (hereafter ‘Yosemite’) from 1992–2010. Predicting toad occupancy patterns and hotspots at the scale of Yosemite is a daunting task because the park is more than 3000 km^2^ in size with thousands of habitat patches that are potentially occupied by toads. *A* *. canorus* site occupancy data from the last two decades suggests that this species has experienced broad declines across its range during the past century, and this once common species is now Proposed for protection under the Endangered Species Act as Threatened and is a California Species of Special Concern [16–19].

*A* *. canorus* typifies the situation for many rare species that occupy patchy habitat: detailed temporal data on colonization-extinction rates are not available to develop a dynamic patch-occupancy model, and sampling gaps in the landscape preclude using characteristics of neighboring patches as predictors of species presence (to infer the role of dispersal [9,20]:). In our modeling approach, we considered the distribution of *A* *. canorus* breeding habitat to be a network of discrete meadow patches linked by dispersal. To describe the habitat characteristics of each of Yosemite’s >2,500 meadows, we developed coarse-scale environmental and landscape (i.e., habitat) variables that we could characterize using remotely sensed data. Our primary focus was to accurately predict the distribution of *A* *. canorus* breeding meadows to: a) estimate the total number of breeding meadows within Yosemite, b) identify breeding ‘hotspots,’ or clusters of high quality breeding meadows in the park, c) rank individual breeding meadows by their potential contribution to breeding at the park scale, and d) improve the efficiency of future field survey efforts for model refinement. The resulting model provides an adaptive management framework that can be applied broadly to other patchily distributed species.

### Study System

*A* *. canorus* occurs between the southern portion of the Lake Tahoe Basin in the north to headwaters of the Kings River in the south between 1,980 m (6,500 ft) and 3,414 m (11,200 ft). The historic range of the species includes six National Forests and two National Parks, with approximately one-third of the historic range in Yosemite [21].

*A* *. canorus* breeding starts in late spring just after snowmelt when adults congregate at seasonal pools, shallow water, the margins of lakes and ponds, and slow moving streams most often associated with meadows. The larvae develop within weeks in ephemeral aquatic habitat and metamorphic toads emerge onto surrounding meadow habitat for cover and foraging. Survey data suggest some sites demonstrate considerable variation in year to year breeding while others are relatively consistent [22]. Natural history of the species associated with the post-metamorphic life stages (i.e., juveniles and adults) is less well understood but in addition to meadows they use surrounding upland habitats [23]. The seasonal movements of juveniles and adults may be limited to ca. 1.25 km from breeding sites [24]. Telemetry data on *A* *. canorus* movement patterns suggest that individuals show relatively high site fidelity at the meadow scale, but not necessarily the individual breeding pond scale [24].

Site occupancy data suggest that *A* *. canorus* has disappeared from 47–69% of the sites where it occurred historically, and remaining populations appear to be more isolated and consist of a small number of breeding adults [22,25–27]. While it is not certain what suite of factors are leading to the decline, potential causes may include airborne pesticides or other environmental toxins, infectious disease, climate change, or other habitat modifications associated with anthropogenic uses [17,27,28]. While non-native fish have been shown to play an important role in the decline of another amphibian species, *Rana* *muscosa* , in Yosemite [9,29–33] they are likely not an important driver of *A* *. canorus* decline [30,34].

## Methods

### Amphibian field surveys and data sources

We focus on meadows as the scale for analysis and prediction of *A* *. canorus* breeding habitat. A meadow layer was created in ArcGIS based on the current vegetation map for Yosemite [35]. This Yosemite vegetation map was produced from over 1600 color infrared aerial photographs taken in 1997, and has a minimum mapping unit of 0.5 ha. Meadow vegetation polygons were identified from the map by selecting four vegetation classes characteristic of meadows in Yosemite: 1) “Semi-Permanently to Permanently Flooded Meadow”; 2) “Intermittently to Seasonally Flooded Meadow”; 3) “Shorthair Sedge Herbaceous Alliance”; 4) “Willow spp./Meadow Shrubland Mapping Unit.” Immediately adjacent meadow vegetation polygons or those separated by a mapped stream were joined to form a single ‘Contiguous Meadow Polygon,’ and used as the focal meadow unit. For each Contiguous Meadow (hereafter, ‘meadow’), the proportion of that polygon comprised of each of the four meadow vegetation types was calculated. Sites that were either 100% “Willow spp./Meadow Shrubland Mapping Unit” or 100% “Shorthair Sedge Herbaceous Alliance” were excluded. In the case of the former, these polygons did not meet our meadow definition: “An ecosystem type dominated by herbaceous species that use surface water and/or shallow ground water, and where woody species may be present and locally dense but not dominant at the meadow scale”. Additionally, preliminary analyses of existing survey data suggested this meadow type is not used by *A* *canorus* for breeding. In the case of meadow polygons comprised of 100% Shorthair Sedge Herbaceous Alliance further inspection of air photos and field visits suggested these were very dry meadows with low likelihood of containing ponded breeding habitat. We excluded non-suitable habitat types prior to surveys instead of including variables describing these habitat types in our model in an effort to reduce the number of potential explanatory variables in our model (see models below) and to reduce the number of sites needing to be surveyed. The final GIS layer in our analysis was comprised of 2,558 meadows.

We synthesized all available *A* *. canorus* survey data from 1992–2010 to identify meadows where *A* *. canorus* breeding was detected, and meadows surveyed but where no *A* *. canorus* breeding was detected. Our three main survey data sets included “Fellers” (1992-2009) (G.M. Fellers unpublished data), “Knapp” (2000-2001) [30], and “NPS/USGS” data collected during this study (2009-2010). All of these independent efforts used almost identical amphibian visual encounter survey methods [30,36,37], but differed in the years sampled and landscape-level sampling strategy. The Fellers’ surveys targeted mapped lakes, ponds, streams, and meadows; and Knapp censused all mapped lakes, ponds, streams, meadows and marshes in the park. Both efforts also surveyed unmapped lakes, ponds, and wet meadow habitat encountered during site visits, but neither had access to the detailed 2007 meadow layer described above. The NPS/USGS focused exclusively on meadows/marshes from this GIS layer because many were previously unmapped and thus not explicitly targeted in prior surveys. Of the 2,558 meadows derived from the vegetation map, Fellers and Knapp crews surveyed 898 meadows between 1992 and 2009, and the NPS/USGS crews surveyed 436 additional meadows (never before surveyed by Fellers or Knapp) in 2009 and 2010. During all surveys the number of *A* *. canorus* of all life stages (adults, juveniles, tadpoles, egg masses) was recorded. Data from the Fellers and Knapp surveys support the focus on meadows as important breeding habitat for *A* *. canorus* . Of all the water bodies they surveyed, over 95% of those where *A* *. canorus* breeding was detected were in, or within 100 m of, a meadow derived from the park vegetation map. Only four breeding observations were >100 m from a mapped meadow and thus were not included in our analysis due to lack of any environmental data associated with those locations.

To evaluate park-wide patterns of *A* *. canorus* breeding and to account for inherent geospatial recording differences among datasets, we assigned any survey effort that took place within 100 m of a meadow to that meadow. Thus, for each meadow for years 1992-2010 we recorded whether *A* *. canorus* was detected or not, as well as whether there was a breeding population of toads. We counted as “breeding” only those meadows with some toad life stage other than adult (i.e., a life stage indicative of recent breeding: egg masses, tadpoles, or young-of-year), and only six meadows registered adults without breeding. All data were collapsed across years, and a meadow with recorded breeding in any one year was classified as a “breeding meadow.” In all, our dataset on meadows that have been surveyed for *A* *. canorus* spans 18 yrs from 1992–2010, and of the 2558 meadows in the park 1344 have been surveyed. Breeding was documented in 179 of the surveyed meadows. All survey data reported here were purely observational and were conducted within Yosemite and permitted by the National Park Service (Study #’s: YOSE-00383, YOSE-00011, YOSE-00016).

### Environmental data

To describe and predict the presence or absence of *A* *. canorus* within meadows, we amassed available remotely sensed and/or geospatial data on multiple biotic and abiotic variables thought to be important to *A* *. canorus* breeding ecology. All environmental and spatial predictors of *A* *. canorus* breeding were compiled at the meadow scale. These included: a) coarse meadow vegetation classes, b) meadow landscape attributes, c) satellite derived snow covered days and melt dates for each meadow, d) modeled mean monthly estimates of meadow temperature and precipitation, and e) satellite derived estimates of summer wetness and aboveground green biomass (Table 1).

**Table 1:** Explanatory variables used in the General Linear Models and General Additive Models chosen from the full set because they were not highly correlated (r < 0.9).

**Variable**	**Description**
*ElevationCentroid*	Elevation of the meadow centroid
*MaximumSlope*	Maximum terrain slope along the shortest path to the nearest meadow
*VegClassDiversity*	Shannon Diversity index of mapped vegetation classes within each meadow polygon. This measure combines the number of vegetation polygons of each type and their aerial extent.
*AvgAnnualWetness*	Inter-annual average (1986-2006) Tasseled Cap Wetness, averaged within each year over all 30m LANDSAT pixels within the meadow.
*SDAvgAnnWetmess*	Inter-annual standard deviation (1986-2006) of the Tasseled Cap Wetness, averaged within each year over all 30m LANDSAT pixels within the meadow.
*AvgSDAnnWetmess*	Inter-annual average (1986-2006) of the spatial standard deviation in Tasseled Cap Wetness across LANDSAT pixels within the meadow.
*AvgAnnNDVI*	Inter-annual average (1986-2006) Normalized Difference Vegetation Index, averaged within each year over all 30m LANDSAT pixels within the meadow.
*SDAnnAvgNDVI*	Inter-annual standard deviation (1986-2006) of the Normalized Difference Vegetation Index, averaged within each year over all 30m LANDSAT pixels within the meadow.
*AvgSDAnnNDVI*	Inter-annual average (1986-2006) of the spatial standard deviation in Normalized Difference Vegetation Index across LANDSAT pixels within the meadow.
*SnowP50.Mean*	Inter-annual average (2002-2007) proportion of days in the water year that the meadow had >50% snow covered area (estimated from daily MODIS).
*SnowP50.SD*	Inter-annual variability (2002-2007) in snow-covered days, measured as the standard deviation among water years in the proportion of days with >50% snow covered area (estimated from daily MODIS).
*MeltDate.Mean*	Inter-annual average (2002-2007) of the first date after April 1^st^ that the meadow had <25% snow covered area (estimated from daily MODIS)
*MeltDate.SD*	Inter-annual standard deviation (2002-2007) of the first data after April 1^st^ that the meadow had <25% snow covered area (estimated from daily MODIS)
*MeanPrecip*	Inter-annual average (1980-1997) of the mean monthly precipitation for the meadow estimated from Daymet.
*SDAvgTemp*	Inter-annual standard deviation (1980-1997) of the mean monthly precipitation for the meadow estimated from Daymet.

The different models varied in details of variable selection, coefficients, and functional forms, but the rank-estimates of breeding probabilities predicted by all models were highly correlated (Spearman’s correlation > 0.95 for all comparisons). Our focus in this study is on prediction of *A* *. canorus* breeding occupancy (and the management consequences of those predictions), rather than interpretation of model coefficients. See the Methods for more details on how these variables were derived.

#### Vegetation

The vegetation types within each meadow (percent meadow area occupied by each of four vegetation classes) were derived from the Yosemite vegetation map described above. This was condensed to a Shannon Diversity summary statistic (i.e., diversity of vegetation classes) by combining the number of meadow vegetation polygons of each type and their area:

$$H=\sum_{i=1}^{V}-[(V_{i}*ln(V_{i})]$$

where V_i_ is the fraction of the area of the entire meadow made up of vegetation class *i*.

#### Meadow Landscape Attributes

Meadow elevation was estimated at the centroid using a 10m Digital Elevation Model (DEM), and the Maximum Slope to reach the nearest meadow was estimated along the least cost path from the focal meadow to the closest edge of the nearest meadow. The distribution of meadows among park watersheds was described by assigning each meadow centroid to a watershed defined by the CalWater 2.2.1 watershed planning units (http://frap.cdf.ca.gov/data/frapgisdata/download.asp?rec=calw221).

#### Snow Covered Area

We used available estimates of daily fractional Snow Covered Area (SCA) that were derived from 500 m NASA Moderate Resolution Imaging Spectroradiometer (MODIS) satellite imagery [38,39]. These data included complete water years (1 October through 30 September of the following year) for 2002-2007 spanning a range of dry and wet years. We calculated the proportion of each water year during which the meadow had greater than 25% and 50% SCA, and the ‘spring melt out date’ as the first day after 1 March on which meadow SCA was less than 25%. These SCA metrics were highly correlated among years, so we used in our analysis the mean and standard deviation (from 2002–2007) of the proportion of snow days (> 25% and >50% SCA) and the mean and standard deviation of the spring melt out date.

#### Precipitation and Temperature

We used Daymet 1 km climate data (http://daymet.ornl.gov) to estimate for each meadow polygon the monthly mean, maximum, and minimum temperature and precipitation from 1980–1997. To characterize average climatic conditions for each meadow we summarized the data as the 18 year average and standard deviation of monthly temperature and precipitation. Summary statistics for monthly maximum and minimum temperature and precipitation were excluded because they were highly correlated with the means. Mean temperature was dropped from the analysis because it was highly correlated with meadow elevation (r = -0.98), which was retained.

#### Wetness and Greenness

We used publicly available 30 m Landsat Thematic Mapper multi-spectral images (LANDSAT) to estimate the inter-annual summer wetness and standing green biomass for each meadow using the Tasseled Cap Wetness and Normalized Difference Vegetation Index (NDVI), respectively [40–42]. Necessary geometric and radiometric corrections were conducted before applying the Tasseled Cap and NDVI transformations. One Landsat scene from mid-July, mid-August, and mid-September for each year between 1986 and 2006 was selected based on image quality and cloud cover. From these data we used zonal statistics to calculate the mean and standard deviation of Wetness and NDVI across all 3 months of the growing season, and then calculated the mean and standard deviation of summer Wetness and NDVI, as well as the average within-meadow standard deviation (i.e., spatial variability in Wetness and NDVI), across all 20 years. These metrics are meant to help distinguish relative differences among meadows in context of meadow specific hydrologic regimes: a) meadows that are consistently wetter or drier on average among years, b) meadows where wetness and productivity are highly sensitive to inter-annual variation in climate versus meadows that are not, and c) meadows heterogeneously wet versus homogeneously wet.

### Predictive Models

While we reduced the number of explanatory variables by removing ones that were highly correlated (r > 0.9), multicollinearity among the remaining predictors can create problems with interpreting model coefficients. Thus we explicitly focus on prediction and avoid interpreting the ecological significance of individual model coefficients. We evaluated six models against *A* *. canorus* data explicitly withheld for testing. All six models generated very similar predictions (see below) despite variation in the details of variable selection and in the shapes of the predictive functions. These patterns also suggest there was no basis for interpreting model coefficients and support our focus on prediction and the management applications of those predictions. Our intended scale of inference is Yosemite National Park. All analyses were conducted using R version 2.12.0 [43].

The models included: 1) “GLMfull”: a general linear model with a logit link function assuming binomial errors (Library “stats”, function “glm”) using all covariates listed above that were not highly correlated; 2) “GLMstep”: a GLM with Akaike Information Criteria (AIC) model selection to choose the most parsimonious combination of explanatory variables using both forward and reverse model selection (Library “MASS”, “stepAIC” function); 3) “GAM”: a non-smoothed General Additive Model that accommodates non-linear relationships (Library “gam”, function “gam”); 4) “GAMs”: a GAM where the non-linear functions are allowed to be smoothed (Library “gam”, function “gam”); 5) “GAMstep”: a GAM where each predictor can be included smoothed or un-smoothed using forward and reverse stepwise AIC model selection (Library “gam”, function “step. gam”); and finally 6) “100subGLM”: this GLM was built in response to the highly skewed prevalence ratio of absence: presence data of nearly 7:1. While prevalence ratios in this domain do not necessarily lead to poor model performance [44] we investigated whether balancing prevalence ratios would create better predictive performance in our particular dataset. We ran the GLMstep 100 times on subsamples of the dataset that included equal numbers of presence and absence meadows. In all cases we retained all breeding meadows in the training dataset, and for each of the 100 models we randomly selected the same number of absence records from the training data. On average, this meant each absence record was included in 16 different models. For each of the 100 subsample datasets we created a standard GLMstep model and calculated the average model prediction across all 100 models. AIC, which we used to select models in several of the procedures detailed above, is a criterion used to measure the relative goodness of fit of a statistical model, and weighs the trade off between accuracy (measured as the maximum likelihood of the model fit to the data) and complexity (measured as the number of parameters) of any given model [45].

**Figure 1:**
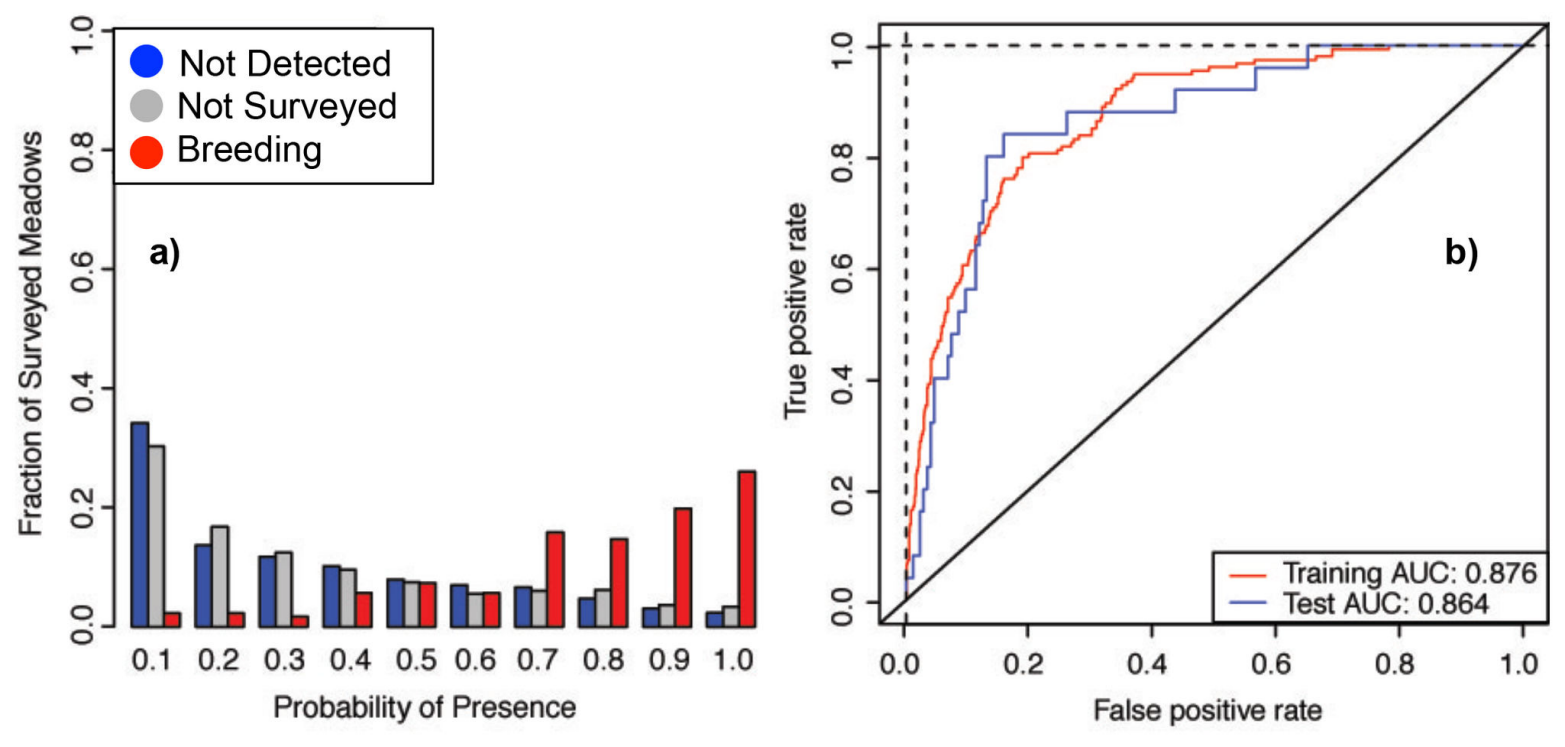
Performance of the “Environmental Model.” a) The distribution of probabilities for the three classes of data for the Environmental Model (see Methods: “100subsampleGLM”). Data are combined from all surveys from 1996–2010. b) ROC plot for the training and test data. The red line shows the fit of the model to the training data, while the blue shows the fit relative to the test data. The curves represent those drawn from the average probabilities across 100 models.

The rank-estimates of breeding probabilities predicted by all models were highly correlated (Spearman’s correlation > 0.95 for all comparisons), and all models had ~80% or more true presences and true absences when tested against observed breeding patterns (see ‘Training and testing the models’). For the rest of the analysis we focus only on the 100subGLM (hereafter “Environmental Model”) — with the primary reason being that it was the most effective model in terms of identifying true presences and true absences (Table 2) in a test dataset. This subsample approach provided a distinct delineation of presence probabilities spanning the full range from 0 to 1 (Figure 1). We use this “Environmental Model” in all subsequent network extensions of our distribution modeling.

**Table 2:** AUC scores, Maximum Discrimination Threshold (MDT) probabilities (the model probability threshold that maximizes both true presences and true absences), and the true presence and true absence rate for the three General Linear Models, three General Additive Models, and the Network-Boosted GLM.

**Purpose**	**Model**	**Metric**	**All Data**	**Train**	**Test**
**Predictive**	**GLM Full**	AUC	.	0.88	0.86
		MDT	.	0.16	0.13
		%True Presence	.	81	80
		%True Absence	.	80	82
**Predictive**	**GLM Step**	AUC	.	0.87	0.86
		MDT	.	0.16	0.12
		%True Presence	.	79	80
		%True Absence	.	80	79
**Predictive**	**100 Sub GLM**	AUC	.	0.88	0.86
		MDT	.	0.56	0.53
		%True Presence	.	80	84
		%True Absence	.	80	84
**Predictive**	**GAM**	AUC	.	0.88	0.86
		MDT	.	0.16	0.12
		%True Presence	.	81	80
		%True Absence	.	80	82
**Predictive**	**GAM Smooth**	AUC	.	0.91	0.87
		MDT	.	0.18	0.11
		%True Presence	.	84	80
		%True Absence	.	84	79
**Predictive**	**GAM Step**	AUC	.	0.90	0.84
		MDT	.	0.16	0.11
		%True Presence	.	81	80
		%True Absence	.	81	80
**Potential Influence of Dispersal**	**Network-Boosted GLM**	AUC	0.864	.	.
		MDT	0.88	.	.
		%True Presence	78	.	.
		%True Absence	78	.	.

The latter includes contributions from nearby meadows (and their neighbors, neighbors of neighbors, etc) to explore the potential influence of dispersal. All the others models only include variables intrinsic to the meadow and were used to predict observed breeding based on meadow covariates (See Methods for more model descriptions).

### Training and testing the models

To test the predictive ability of our models, we trained and tested our models with two different sets of data. The test dataset was comprised of 177 randomly chosen meadows without breeding, and 25 randomly chosen breeding meadows. The numbers of presence and absence meadows in the test dataset were chosen for two purposes. First, given the very small sample of meadows with breeding, we did not wish to remove too many breeding meadows from our training data. Second, we wanted to match the ratio of all presence to absence data in our entire dataset (179 presence, 1165 absence, ~13%). After removing these randomly chosen meadows for testing purposes, we were left to train our model with 154 presence meadows and 988 absence meadows.

The predictive performance of each model was assessed using several criteria, including visual inspection of receiver operator characteristic (ROC) plots and area under the curve (AUC) statistics [46], and a maximum discrimination threshold (MDT). To determine MDT we iteratively set a probability threshold from 0 to 1 in increments of 0.01. For each threshold any meadow with a probability of breeding greater than the threshold was predicted to contain a breeding population, and below that probability the meadow was not considered a breeding meadow. For both the training and the test datasets we calculated the fraction of the observed presence data correctly predicted and subtracted from that the fraction of the observed absence data correctly predicted, and determined the MDT as that which minimized the difference.

### Network analyses

Using the Environmental Model described above, we obtained a predicted probability of *A* *. canorus* breeding at all meadows in Yosemite. We call this probability for any given *i*th meadow P_*ii*_ (in other words, the influence of meadow *i* on itself). P_*ii*_ can be thought of as a meadow’s probability of *A* *. canorus* breeding independent of any network properties that meadow might have.

We know dispersal is an important ecological phenomenon generally, and specifically in our system [19,21,23]. Statistical approaches that allow dispersal information to be included in regression models either use autocovariates constrained by a dispersal kernel [9] or conditional autoregressive spatial models [47]. An autocovariate in this sense describes the presence of breeding at a given meadow as a function of breeding at neighboring meadows – and would be a reasonable approach if all meadows in Yosemite NP had been surveyed for *A* *. canorus* breeding. However, this approach is problematic because in our data set many meadows are surrounded by unsurveyed meadows with no information about breeding status. The purpose adding a spatial network analysis was to examine the role that the potential dispersal network plays for individual meadow influence on park-wide breeding, given the best fit regression models chosen above. The network analysis attempts to use, rather than improve, the predictive accuracy of those models. Therefore, we incorporated the network component of Yosemite meadows by simulating dispersal dynamics across the meadow network in an ecologically informed manner [48]. This approach goes beyond the statistical autocovariate approaches described above by incorporating information about not just neighboring meadows, but their neighbors, neighbors of their neighbors, and so on. We do this according to the equation:

$$P_{i_{total}}=1-\left[\left(1-P_{i}\right)*P_{ij}\right]$$

where

$$P_{ij}=\prod_{j}\left(1-P_{i}*P_{j}*D_{ji}\right)$$

By this equation, meadow *i*’s overall probability of having toad breeding is a function of P_*i*_ and the influence of all of its *j* neighbor’s P_*j*_ values, weighted by the probability of dispersal from all meadows *j* to *i* (D_*ji*_). We implement D_ji_ as a linear dispersal kernel with a maximum cutoff of 1 km – where probability of dispersal at a distance of 1 km is 0. The maximum known *A* *. canorus* dispersal distance recorded in the field is 1,261 m for a female and 865 m for a male [24,49–52]. We used a linear dispersal kernel because, in the absence of dispersal data to constrain our assumption, we had little reason to use a more complex function (e.g., negative exponential). Thus, P_*ij*_ (when *i* is not equal to *j*) is the probability of dispersal from meadow *j* times the intrinsic environmental probability of toad breeding at meadow *j*, times meadow *i*’s own intrinsic probability. We took the product of all meadow *j* to meadow *i* combinations, after subtracting the weighted probabilities from 1 (the probability of breeding not occurring at the meadow), and allowed meadows with higher intrinsic probabilities to benefit proportionately from immigration in our calculation of 1-P_*i*_. Subtracting everything again from 1 returns the values to the probability of breeding, influenced by immigration from neighboring sites. We then simulated these dynamics across the network until the probability of breeding across sites ceased to change (10 iterations) to obtain updated network-influenced probabilities of breeding for each meadow within Yosemite. Using the product of probabilities serves to normalize the iterative contribution of nearest neighbors in the network simulation to a value between 0 and 1. We call this model the ‘Network-Boosted GLM’ – and it incorporates the influence not only of nearest neighbors, but also neighbors of neighbors, etc. A key reason for our approach was to calculate the relative improvement each meadow experienced due to dispersal effects as “Normalized Network Improvement” or NNI. NNI is defined as:

$$NNI_{i}=\frac{P_{i}{}_{{}_{total}}-P_{i}}{1-P_{i}}$$

This equation calculates how much a meadow’s probability of breeding increased relative to the amount it could increase (1 -P_*i*_). For example, if the intrinsic P_*i*_ is 0.60, and the Network-Boosted probability (P*i*_*total*_) is 0.80, then the NNI is 0.50. The maximum possible value of NNI is one.

Using this approach we assessed the influence of every meadow on the total breeding structure of Yosemite. The summed probability of breeding across the entire Yosemite study site is:

$$ExpBreed_{T}=\sum P_{i}{}_{{}_{total}}$$

The influence of each meadow was calculated by iteratively disconnecting each node by changing its distance to all other meadows to above the cutoff threshold in the dispersal kernel. Thus, meadow *i* still has the same P_i_, but does not contribute to any other meadow’s probability. We then calculated

$$ExpBreed_{i}=\left(\sum_{N-i}P\right)+P_{i}$$

where meadows i’s probability is only equal to P_i_. In this way we ranked every node by its system-wide network influence (G_*i*_), which is calculated as

$$G_{i}=ExpBreed_{T}-ExpBreed_{i}$$

The bigger a meadow’s G_i_ value, the more influential it is in our dispersal simulations. This provided us with an estimate of the global network influence of every meadow on the overall number of breeding meadows in Yosemite.

## Results and Discussion

### GLM Results

Overall, the Environmental Model (i.e., no network consideration) predicted 84% of *A* *. canorus* true presence and absence meadows across the landscape in our test dataset (Figure 1a, Table 2). This ability to discern breeding and non-breeding habitat is confirmed with AUC scores near 0.9 (Figure 1b), a common criteria for model support [46]. Finally, the consistency in rank estimates of breeding probabilities across all model approaches (Spearman’s correlation > 0.95 for all comparisons) lends support to using these model predictions to prioritize unsurveyed meadows for future assessment and in the development of the network model.

Our Environmental Model (Figure 1) predicts that most unsurveyed meadows have a low probability of *A* *. canorus* breeding. The MDT for the testing dataset – which maximizes both true presences and true absences of *A* *. canorus* on a set of data the model has not seen before- is 0.53. This threshold we predicts that 84% of all unsurveyed meadows above a probability of 0.53 will have breeding, while 16% of meadows below the threshold should also contain breeding populations. There are 277 unsurveyed meadows with probabilities higher than the threshold (277 * 0.84 = 233 breeding meadows) and 937 unsurveyed meadows below the threshold (937 * 0.16 = 150), resulting in a predicted total number of potential breeding meadows within YNP to be 562 (179 known + 383 unsurveyed) of 2,558 total meadows. This estimate is conservative given that detectability of *A* *. canorus* , even if it is present, is not perfect (see below).

### Meadow ‘Environmental Quality’ vs. ‘Network Quality’

**Figure 2:**
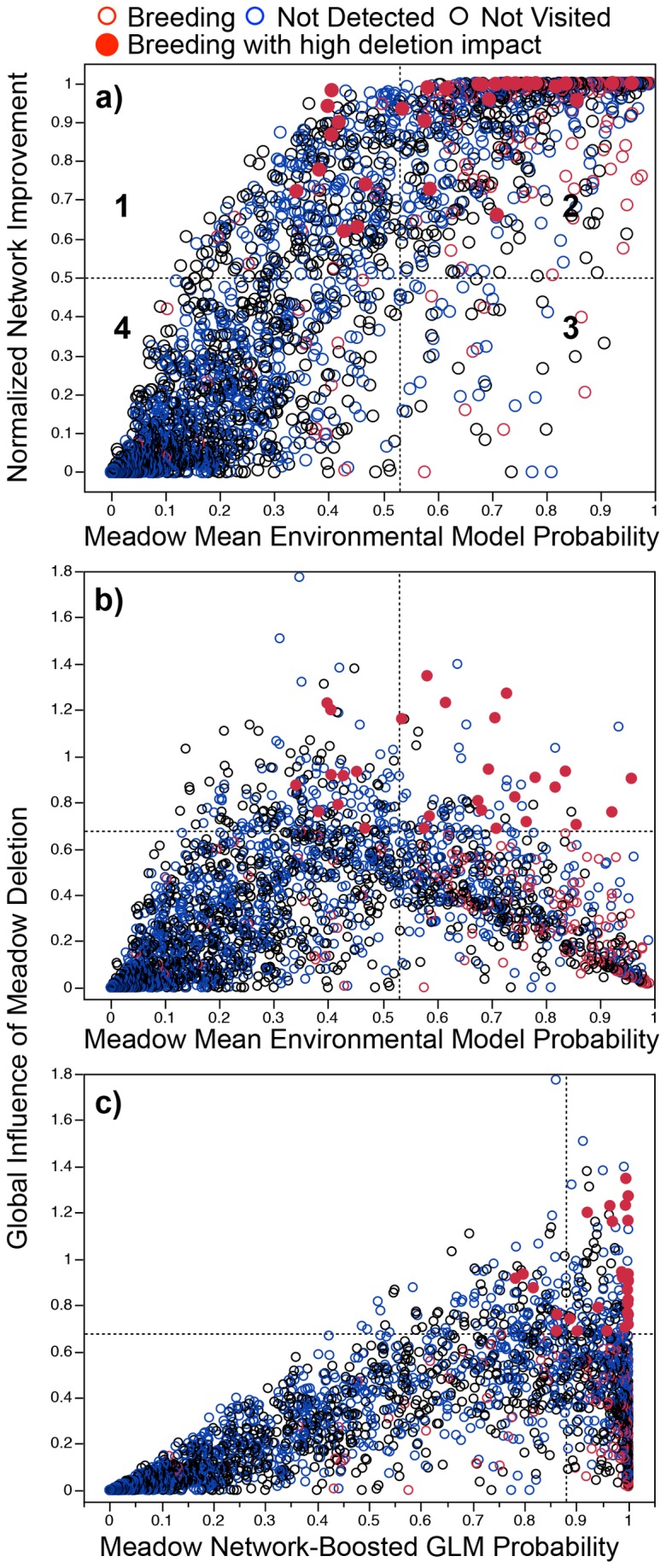
Disentangling intrinsic meadow ‘environmental quality’ from its spatial ‘network quality’ with respect to predicted *A* *. canorus* breeding. a) Normalized Network Improved breeding probabilities ((Network Boosted probability – Environmental Model probability) / (1 – Environmental Model probability)) for each meadow as a function of the mean Environmental Model probability. Symbol colors represent field survey results (Breeding detected (red), Breeding not detected (blue), and Meadow not visited (black). b-c) Total Influence (G_*i*_) of meadow deletion (the decrease in park-wide breeding probability summed across all meadows after deleting that meadow from the network) as a function of the Environmental Model and the Network-Boosted GLM probabilities. Horizontal dotted lines indicate the 90^th^ percentile of meadow deletion impact, and solid red symbols are the known breeding meadows within that 90^th^ percentile. Vertical dotted lines in all panels represent the threshold probability that maximizes both true positives and true negatives predicted by the model.

The GLM model results reported above include only information pertaining to a discrete meadow, and ignore the fact that these meadows are spatially clustered in ways that might make it more or less difficult for a given meadow to be colonized by breeding individuals - including colonization that could “rescue” populations in patches from local extirpation. Overall, the rank order of meadow breeding probabilities is similar between our ‘Network-Boosted GLM’ (where the intrinsic probabilities are ‘boosted’ by the quality of neighboring meadows, as well as that of their neighbors – see Methods) and the Environmental Model (Spearman’s rank correlation = 0.96). However, comparing the two approaches allows us to distinguish the estimated ‘environmental quality’ of a meadow (i.e., coarse environmental attributes conducive to breeding) from its estimated ‘network quality’ (i.e., meadow neighborhood conditions conducive to colonization) (Figure 2a). We defined ‘high’ environmental quality to be greater than the Maximum Discrimination Threshold of the GLM (0.53), and ‘high’ network quality to be greater than 50% Normalized Network Improvement. In Yosemite, 76% of *A* *. canorus* detections (red open or closed symbols) occurred in meadows that were predicted to have both high environmental quality and high potential connectivity to neighboring populations (Figure 2a, quadrant 2). These meadows could be high priority for management because these clusters of high environmental quality breeding meadows are potentially well connected to each other. Approximately 10% of *A* *. canorus* breeding detections were in meadows with high environmental quality that are isolated from other high quality meadows (Figure 2a, quadrant 3). These meadows could merit further investigation for their potential contribution to genetic diversity or as refugia from disease. These isolated breeding meadows may also be high priority for protection if there is lower potential for natural re-colonization after a local extirpation. Another 10% of meadows had lower predicted environmental quality, but were spatially well suited to be colonized by toads inhabiting nearby higher environmental quality meadows (Figure 2a, quadrant 1). These meadows might be expected to show intermittent breeding or frequent local extinction and re-colonization from toads inhabiting nearby meadows. Only 4% of *A* *. canorus* detections were in meadows with low environmental quality and low network quality (i.e., they are isolated from other high environmental quality meadows) (Figure 2a, quadrant 4). These meadows may merit further investigation to provide a better understanding of the finer-scale habitat attributes within these meadows that are allowing breeding. Including variables that describe these more detailed, within-meadow attributes could improve the accuracy of future modeling efforts.

### Park-Wide Meadow Network Influence

Incorporating information about the meadow dispersal network also offers insights into which meadows contribute the most to *A* *. canorus* breeding at the scale of the entire park. Individual meadows with the biggest deletion influence (see G_*i*_ in Methods) on park-wide breeding are generally not those with the highest intrinsic breeding probability (Figure 2b). For example, the latter tend to be surrounded by other high probability meadows, resulting in most of those falling in quadrant 2 of Figure 2 (if they were isolated or surrounded by low probability neighbors, they would fall in quadrant 3). Instead, breeding meadows with intermediate intrinsic environmental quality but high connectivity appear to have the biggest park-wide impact (Figure 2a and 2b, solid red symbols). Maximum meadow deletion impact is better predicted by the network-boosted model probability (Figure 2c) than by the intrinsic environmental (GLM) breeding probability (Figure 2b). However, not all breeding meadows with high network boosted probability have high deletion impact (Figure 2c). For example if a meadow is surrounded by many meadows with high intrinsic breeding probability, deletion of that meadow will likely not have a large impact on the breeding probability of the neighbors.

It is important to note that our conclusions about meadow network impact are based on a network structure derived from available adult movement data and the simplest possible dispersal kernel in the absence of other data. The extent to which this approach approximates actual toad dispersal patterns remains largely unknown. Our modeling framework can easily accommodate more detailed dispersal data collected in the future. Additional analyses, however, suggest that increasing the dispersal distance 5-fold or changing the shape of the dispersal kernel to a beta distribution do not change the outcomes reported here.

### Landscape Scale Patterns

**Figure 3:**
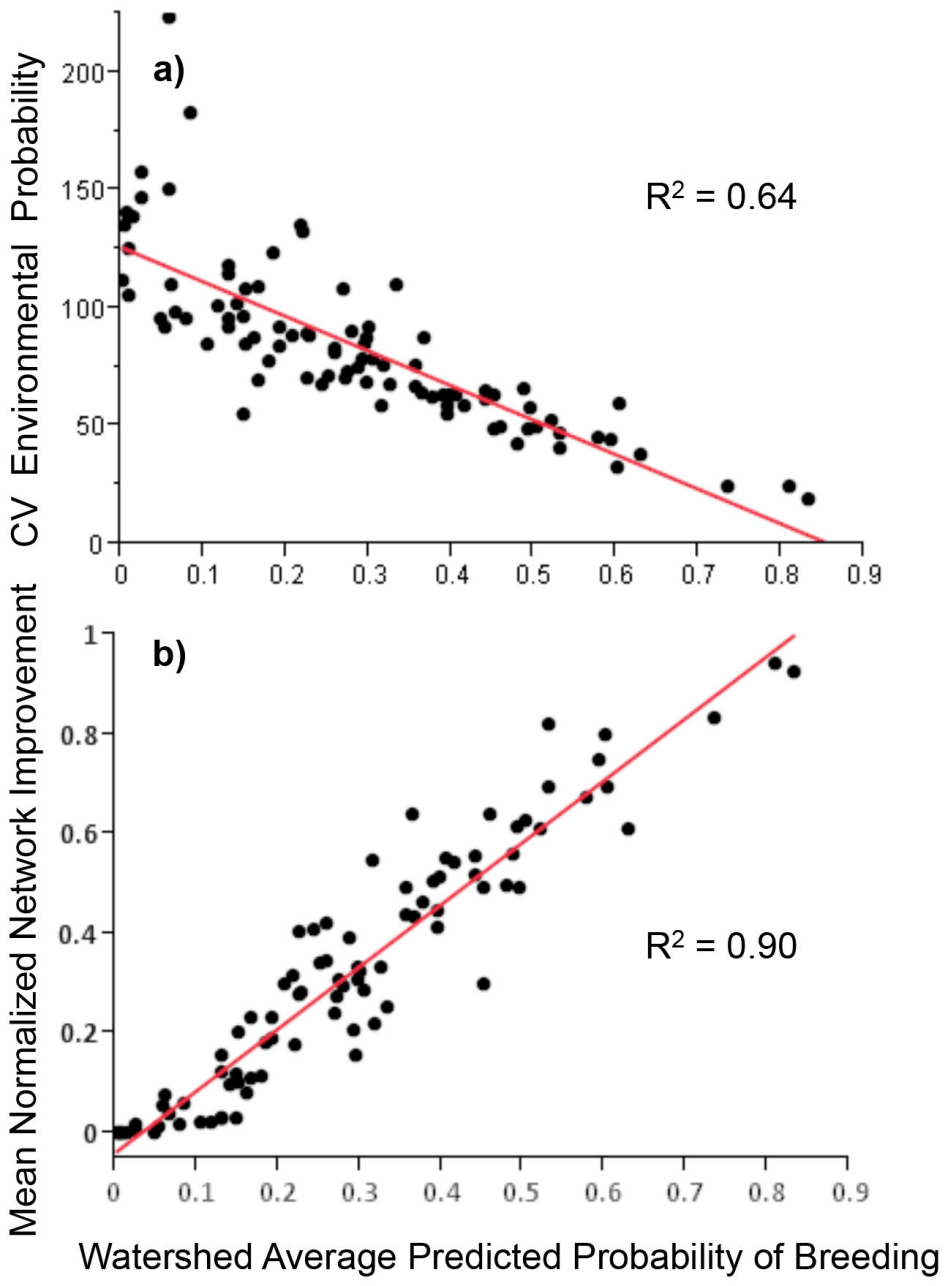
Watershed scale patterns of predicted breeding. a) Watersheds with high mean predicted probability of *A* *. canorus* breeding (Environmental Model) tend to have low coefficient of variation (CV) in breeding probabilities among meadows within the watershed. b) Watersheds with high mean breeding probability across also show high normalized network improvement in the predicted breeding probabilities due to spatial clustering of ‘intrinsically good’ meadows. ‘Normalized network improvement’ is the proportional increase in Environmental Model predicted probability relative to the maximum improvement possible.

**Figure 4:**
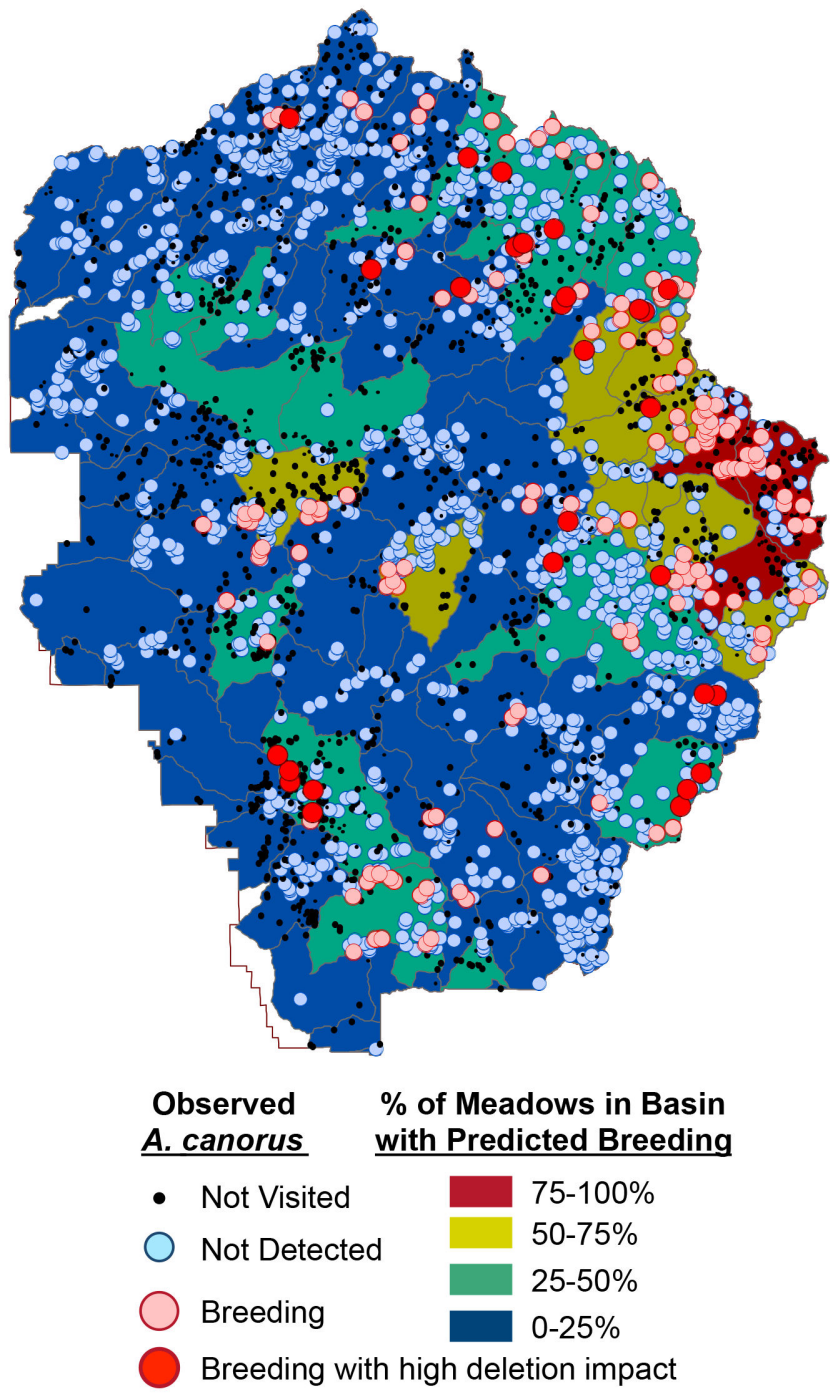
Observed and predicted distribution of meadows and watersheds with *A* *. canorus* breeding in Yosemite National Park. The watersheds (Cal 2.2.2 watershed planning units) are colored by the percent of meadows within the basin that are predicted by the Network Boosted GLM model to have breeding. The symbols are centroids for all 2,558 contiguous meadows in the park. Pink and/or red symbols indicate a meadow with observed *A* *. canorus* breeding at least once between 1992–2010. Blue symbols were visited at least once with no *A* *. canorus* detected. Small black dots are meadows that have not been visited. Solid read symbols are meadows with breeding that are in 90^th^ percentile of impact on park-wide breeding probability if deleted. Note that meadows with high deletion impact are not in the watersheds with highest proportion of ‘good’ meadows.

Taken together, these patterns suggest Yosemite meadows that are ‘environmentally good’ for *A* *. canorus* breeding also tend to be spatially clustered and connected (i.e., they have high ‘network quality’). In Figure 3 we plot the mean predicted Environmental Model probability of breeding across all meadows within each watershed unit (see Methods) against the coefficient of variation in probability among meadows within that watershed. Watersheds with high mean meadow probability of breeding tend to have low variation among meadows (Figure 3a). Similarly, those watersheds with the highest average breeding probability across meadows are also the watersheds with the highest normalized network improvement due to the influence of nearby high probability meadows (Figure 3b). Together these trends suggest that intrinsically high quality meadows tend to be clustered within watersheds. Watersheds with the highest proportion of meadows with predicted *A* *. canorus* breeding are themselves aggregated at the eastern edge of the park (Figure 4). Interestingly, known breeding meadows with high deletion impact on park-wide summed breeding probability (Figure 4, solid red symbols) are not in the watersheds with the highest proportion of meadows with predicted breeding (Figure 4, red watersheds). They tend to occur in watersheds where only 20-50% of the meadows are predicted to have breeding (Figure 4). The spatial aggregation of both observed and predicted high probability *A* *. canorus* breeding meadows within Yosemite suggests that conservation of *A* *. canorus* could be optimized at the watershed scale. This approach could target a) large, connected, clusters of meadows with high environmental quality with the potential to support a resilient meta-population, b) smaller clusters of meadows that may contribute most to the broader park-wide *A* *. canorus* distribution, and c) isolated *A* *. canorus* meadows that may be vulnerable to permanent extirpation. Similarly, this approach could also predict currently unoccupied meadows that may especially valuable for restoration efforts.

## Conclusions

Using the Yosemite Toad ( *A* *. canorus* ) as a case study, we present a network extension of a static species habitat occupancy model that enables inferences about the relative role of intrinsic ‘environmental quality’ vs. ‘network quality’ of habitat patches linked by dispersal. This approach maximizes the use of limited species presence/absence data to estimate spatial processes where extensive colonization-extinction data are not available to parameterize a dynamic patch-occupancy model [3] and where sampling gaps in the landscape preclude the use of spatial autocorrelation in species presence to infer the role of dispersal [9].

Despite the relatively coarse scale of environmental covariates we could gather for all 2,558 meadows in Yosemite, our simple model had ~84% or higher true presence and absence rates when tested against *A* *. canorus* breeding observations. Multiple model approaches (e.g., three GLM’s and three GAM’s) all had similar rank-order predictions of *A* *. canorus* breeding probabilities despite differences in which covariates were included and the shapes of the functions. Three likely sources of prediction error include:

### 1: Lack of detection by field observers

False positives (meadows with predicted breeding but where no breeding was observed) could be the lack of detection rather than the absence of *A* *. canorus* . Data from double surveys of over a five year period (2007-2011) suggest that the probability of detecting *A* *. canorus* if it is indeed breeding is approximately 75% (G. M. Fellers, unpublished data). Field observations suggest that sites visited either very early (during the egg stage) or late (during metamorphosis) likely have lower detectability and may merit re-visiting.

### 2: Annual intermittency in breeding

Even if *A* *. canorus* adults are present in a meadow, they do not necessarily breed every year. Thus a false positive can occur due to lack of breeding that year even if breeding, in general, occurs in that meadow [22]. In our database, while the NPS/USGS crew surveys in 2009 and 2010 focused on visiting previously un-surveyed meadows, each year an average of 152 meadows had a prior survey. On average 42% of those meadows with prior breeding did not have detected breeding in the re-survey, and 17% of those with a prior visit and no detected breeding, but high predicted breeding probability, had breeding detected in the subsequent visit. Further field sampling targeted at meadows with high predicted breeding probability could help better characterize these patterns of intermittency. Based on our model, we would predict higher levels of intermittency for meadows with marginal ‘intrinsic’ environmental quality but high ‘spatial quality’ (i.e., Figure 2a, Quadrant 1).

### 3: Fine-scale habitat characteristics

Even if the coarse-scale environmental and geo-spatial attributes of a meadow are conducive to *A* *. canorus* breeding, whether or not breeding is realized very likely depends on a suite of fine scale aquatic habitat features, such as shallow water with solar exposure that persists long enough for larval development to be completed. Similarly, meadows with low predicted breeding probability that had observed breeding (i.e., ‘false negatives) could be cases where the coarser scale attributes did a poor job of predicting these finer scale habitat features.

### 4: Extirpation of the species

Some meadows with predicted, but not observed, breeding (false positives) could have suitable breeding habitat (based on coarse environmental and spatial predictors) but lack *A* *. canorus* due to local extirpation from anthropogenic stressors. Further data on the spatial distribution of different potential stressors across meadows in the park would be necessary to fully evaluate this hypothesis.

Given these potential sources of error, it is impressive that our simple linear model approach, based on coarse meadow-scale attributes, had ~84% accuracy in predicting meadows with (and without) *A* *. canorus* breeding. Our results suggest that environmental and network ‘quality’ largely coincide for the vast majority of *A* *. canorus* breeding meadows in Yosemite. In other words, meadows with high intrinsic predicted breeding probability tend to be surrounded by other intrinsically ‘good’ neighbors (that also have good neighbors). Outliers from this trend highlight breeding meadows with high individual environmental quality that are very isolated from other high environmental quality meadows (Figure 2a, Quadrant 3) and may merit special conservation attention. Perhaps surprisingly, clusters of meadows with lower predicted environmental quality may contribute disproportionately more to *A* *. canorus* breeding at the entire park scale than do meadows in spatial clusters of high environmental quality meadows (Figures 2b and 4).

By incorporating a spatial network structure into a static species habitat occupancy modeling framework we used minimal occupancy data to estimate the relative importance of the environmental versus network quality of individual habitat patches in a landscape context. This approach can inform management decisions for *A* *. canorus* by predicting: a) breeding ‘hotspots’ in Yosemite where clusters of high quality breeding meadows are potentially linked by dispersal, b) high environmental quality breeding meadows that are isolated from other such meadows, and c) breeding meadows with marginal environmental quality that may critically depend on (or contribute to) the quality of their network neighborhood. These results also help focus the collection of additional data on dispersal, genetics, fine-scale habitat quality, and potential stressors (e.g., disease) to inform the designation of core conservation areas for different management objectives (e.g., meta-population stability, genetic diversity, refugia from disease outbreaks, high impact restoration sites). This network extension of a static species occupancy model may also be useful for predicting source and sink populations for other rare species when sufficient occupancy data or colonization-extinction data are limited.
